# Traditional Chinese Medicinal Herbs for Treating Post‐Ischemic Stroke Cognitive Impairment Insights From Frequency and Association Analysis

**DOI:** 10.1002/brb3.70829

**Published:** 2025-09-02

**Authors:** Yixia Li, Wenqi Huang, Jing Sun, Wenjuan Li

**Affiliations:** ^1^ Department of Encephalopathy Jing'an District Hospital of Traditional Chinese Medicine Shanghai China

**Keywords:** association rule mining, cognitive impairment, frequency analysis, ischemic stroke, medicinal herbs, traditional Chinese medicine

## Abstract

**Background:**

This study analyzes medicinal herbs in Traditional Chinese Medicine (TCM) formulations for post‐ischemic stroke cognitive impairment, identifying key herbs, their interactions, and potential therapeutic mechanisms through systematic review, frequency analysis, and association rule mining.

**Methods:**

Systematic searches were performed across major databases (CBM, CNKI, VIP, Wanfang Data) to find studies on TCM for post‐ischemic stroke cognitive impairment. Inclusion criteria focused on studies reporting TCM formulas and their cognitive effects. Data extraction standardized herb names, and frequency analysis identified high‐frequency herbs (>10%). Association rule mining was done using Python with a minimum support of 10%, a minimum confidence of 50%, and a maximum antecedent size of 5 to uncover key herb associations for therapeutic insights.

**Results:**

A systematic review identified 56 studies on TCM formulas for post‐ischemic stroke cognitive impairment, extracting 114 herbs. Frequency analysis found 27 herbs used in over 10% of formulas, with *Ligusticum chuanxiong*, *Acorus tatarinowii*, and *Salvia miltiorrhiza* being most common. Association rule mining revealed key combinations, suggesting potential synergistic effects in TCM treatments.

**Conclusion:**

This study highlights key traditional Chinese herbs and herb combinations commonly used in clinical practice for cognitive impairment after ischemic stroke. The findings suggest that herb pairs such *as Ligusticum chuanxiong*–*Salvia miltiorrhiza* and *Acorus tatarinowii–Polygala tenuifolia* may exert synergistic effects consistent with both TCM theory and modern pharmacology. These results provide direction for future targeted studies and support the rational formulation of TCM‐based interventions in stroke rehabilitation.

## Graphical abstract

This analysis highlights key herbs and their interactions, suggesting significant implications for further research into their efficacy and safety for stroke rehabilitation.

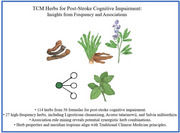

## 1 Introduction

In 2020, the “China Stroke Report” indicated that the prevalence of stroke in China is 1114.8 per 100,000 people, with an annual incidence rate of 246.8 per 100,000 and a mortality rate of 149.49 per 100,000 (Wang et al. 2020; Tian et al. 2022). Approximately one‐third of stroke patients experience post‐stroke cognitive impairment (PSCI), the majority of whom suffer from cognitive impairment following ischemic stroke (Kuchcinski et al. 2017). PSCI can lead to a range of adverse outcomes, such as delayed functional recovery, reduced quality of life, and increased unnecessary economic burden on families and society. Therefore, early prevention, assessment, and timely treatment are crucial for the disease's progression and prognosis.

Current modern medical treatments for PSCI mainly involve interventions targeting stroke‐related risk factors (such as hypertension, diabetes, and hyperlipidemia) and pharmacotherapy aimed at potential pathological pathways. Key medications include cholinesterase inhibitors, such as donepezil (Kim et al. 2020), non‐competitive N‐methyl‐D‐aspartate (NMDA) receptor antagonists like memantine, and other drugs such as nimodipine, nicergoline, and butylphthalide, which have been found to improve cognitive function (Yang et al. 2022; Klamkam et al. 2022; Zhou et al. 2019). However, there is still a lack of large‐scale targeted studies and universally recommended medications.

Traditional Chinese Medicine (TCM) has long recognized and extensively discussed cognitive impairment following ischemic stroke, categorizing it under terms like “forgetfulness,” “dementia,” “idiocy,” and “mental dullness,” with symptoms primarily characterized by cognitive dullness and slow thinking. Recent studies increasingly demonstrate that TCM can significantly improve patients' cognitive functions, behavioral thinking, and daily living abilities (Guo et al. 2023). The effectiveness, safety, and reliability of TCM have become more apparent in recent years, leading to a growing number of documented cases. However, there is still a relative lack of research focusing on the characteristics of TCM prescription patterns for treating this condition.

Therefore, this study aims to analyze the characteristics and patterns of TCM prescriptions for cognitive impairment following ischemic stroke by collecting and applying data mining techniques to clinical research from recent years. This research aims to provide evidence for clinical practice and new drug development in TCM.

## 2 Materials and Methods

### 2.1 Literature Search

The databases searched included the China Biomedical Literature Database (CBM), China National Knowledge Infrastructure (CNKI), VIP Database, and Wanfang Data. Considering that most TCM clinical studies are published in Chinese‐language journals, we focused our search on Chinese databases (CBM, CNKI, VIP, and Wanfang), which comprehensively cover relevant literature. International databases such as PubMed and Web of Science were not included, as they contain limited records on Chinese‐language TCM formulations used in clinical practice. This choice ensured a more accurate and exhaustive capture of relevant prescriptions applied within the Chinese medical system. Keywords used were “post‐stroke cognitive impairment,” “stroke,” “dementia,” and “Traditional Chinese Medicine treatment,” set as subject terms. The search period was from the establishment of the database until June, 2024. A database was constructed based on these searches, with duplicate records removed.

### 2.2 Literature Screening

#### 2.2.1 Inclusion Criteria

Studies were only included if they met the diagnostic criteria outlined in the 2019 Chinese Guidelines for the Diagnosis and Treatment of Vascular Cognitive Impairment issued by the Neurology Branch of the Chinese Medical Association (Chinese Stroke Association Vascular Cognitive Impairment 2024). Additionally, eligible studies were required to include at least 30 clinical cases, a treatment duration of no less than 4 weeks, and a clearly reported TCM prescription containing three or more herbal components. Only those studies employing compound formulas or integrated TCM–Western interventions were retained. Moreover, studies had to report measurable cognitive outcomes, with the treatment group demonstrating superiority over controls in terms of total effective rate (≥ 70% based on TCM symptom score) and/or significant improvements in validated cognitive assessments such as the Mini‐Mental State Examination (MMSE), Montreal Cognitive Assessment (MoCA), and Activities of Daily Living (ADL) scales. To ensure scientific robustness, we also included only studies with clear research design, well‐defined data collection procedures, and appropriate statistical analysis methods.

#### 2.2.2 Exclusion Criteria

(1) Studies with unclear diagnoses or comorbidities. (2) Studies with unclear or incomplete descriptions of medication composition. (3) Studies with different prescription names but identical compositions, counted as a single study. (4) Studies using non‐traditional TCM decoctions, such as health products, external medicines, or injectable drugs.

### 2.3 Database Construction and Data Cleaning

An Excel 2020 database was established for TCM compound formulas. Standardized naming conventions were applied according to the “Thirteenth Five‐Year Plan” textbooks on Chinese Materia Medica published by the China Traditional Chinese Medicine Publishing House. For instance, Jiang Banxia and Fa Banxia were standardized as Banxia. Each herb was assigned a binary value (1 for presence, 0 for absence). Two individuals independently entered the data, followed by review and verification.

We acknowledge that combining different processed forms of certain herbs (e.g., Pinellia) under a single standardized name may introduce variability in pharmacological effects. This limitation was necessary for data harmonization across heterogeneous clinical reports but should be considered when interpreting results involving such herbs.

### 2.4 Data Mining

Frequency analysis was conducted on the usage frequency, properties, meridian tropism, and efficacy of individual herbs. For high‐frequency herbs (usage frequency ≥ 10%), SPSS 18.0 was used for data mining, employing hierarchical clustering based on the properties, meridian tropism, and primary therapeutic effects of the herbs. Python was used to perform association rule analysis and to visualize the network of herbal combinations.

## 3 Results

### 3.1 Literature Screening Results

**FIGURE 1:**
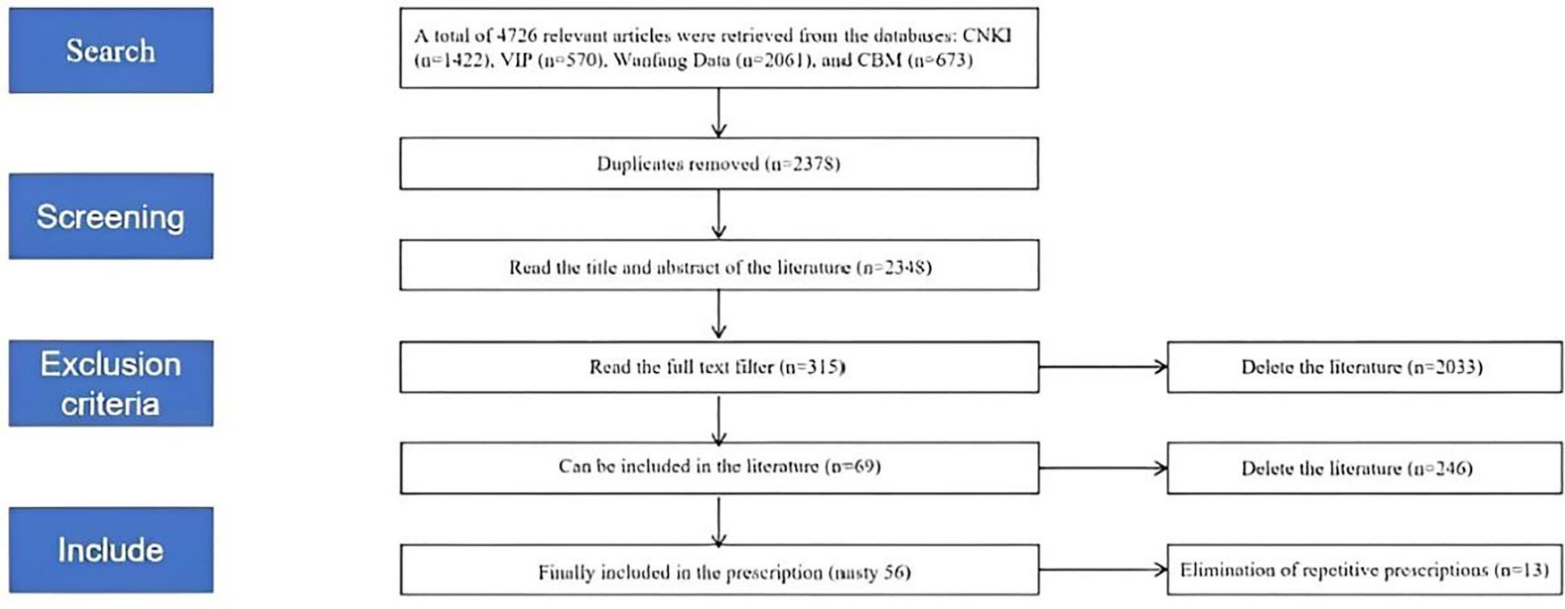
Literature screening process and results.

Two researchers independently conducted literature searches, yielding 4726 relevant studies on the use of TCM for PSCI. Based on inclusion and exclusion criteria, 69 studies met the standards, and after further processing, 56 TCM formulas were identified. The detailed process of database search, literature, and formula screening is illustrated in Figure 1.

### 3.2 Frequency and Frequency Analysis of High‐Frequency Chinese Medicines for Treating Cognitive Impairment Post‐Ischemic Stroke

From the 56 TCM formulas, a total of 114 distinct Chinese medicinal herbs were identified after standardizing their names. These herbs appeared a total of 496 times in the formulas. Among them, 27 herbs were used with a frequency greater than 10%. The top five herbs by frequency were Chuanxiong (*Ligusticum chuanxiong*), Shichangpu (*Acorus tatarinowii*), Danshen (*Salvia miltiorrhiza*), Gancao (*Glycyrrhiza uralensis*), and Honghua (*Carthamus tinctorius*). The frequency and usage rates of high‐frequency herbs are shown in Table 1.

**TABLE 1:** Medicinal herb frequency.

Rank	Medicinal Herb	Frequency	Percentage
1	*Ligusticum chuanxiong*	28	50.00%
2	*Acorus tatarinowii*	27	48.21%
3	*Salvia miltiorrhiza*	24	42.86%
4	*Carthamus tinctorius*	16	28.57%
5	*Angelica sinensis*	16	28.57%
6	*Polygala tenuifolia*	15	26.79%
7	*Glycyrrhiza uralensis*	14	25.00%
8	*Paeonia lactiflora*	14	25.00%
9	*Rehmannia glutinosa*	12	21.43%
10	*Panax ginseng*	11	19.64%
11	*Polygonatum sibiricum*	11	19.64%
12	*Astragalus membranaceus*	10	17.86%
13	*Polygonum multiflorum*	10	17.86%
14	*Gastrodia elata*	10	17.86%
15	*Semen erythrophlei*	9	16.07%
16	*Cornus officinalis*	9	16.07%
17	*Curcuma aromatica*	8	14.29%
18	*Paris polyphylla*	8	14.29%
19	*Poria cocos*	8	14.29%
20	*Panax notoginseng*	7	12.50%
21	*Buthus martensi*	7	12.50%
22	*Hirudo*	7	12.50%
23	*Prunus persica*	7	12.50%
24	*Bombyx mori*	6	10.71%
25	*Pinellia ternata*	6	10.71%
26	*Glycyrrhiza glabra*	6	10.71%
27	*Citrus reticulata*	6	10.71%

### 3.3 Analysis of the Properties and Meridian Tropism of Chinese Medicines for Treating Cognitive Impairment Post‐Ischemic Stroke

**FIGURE 2:**
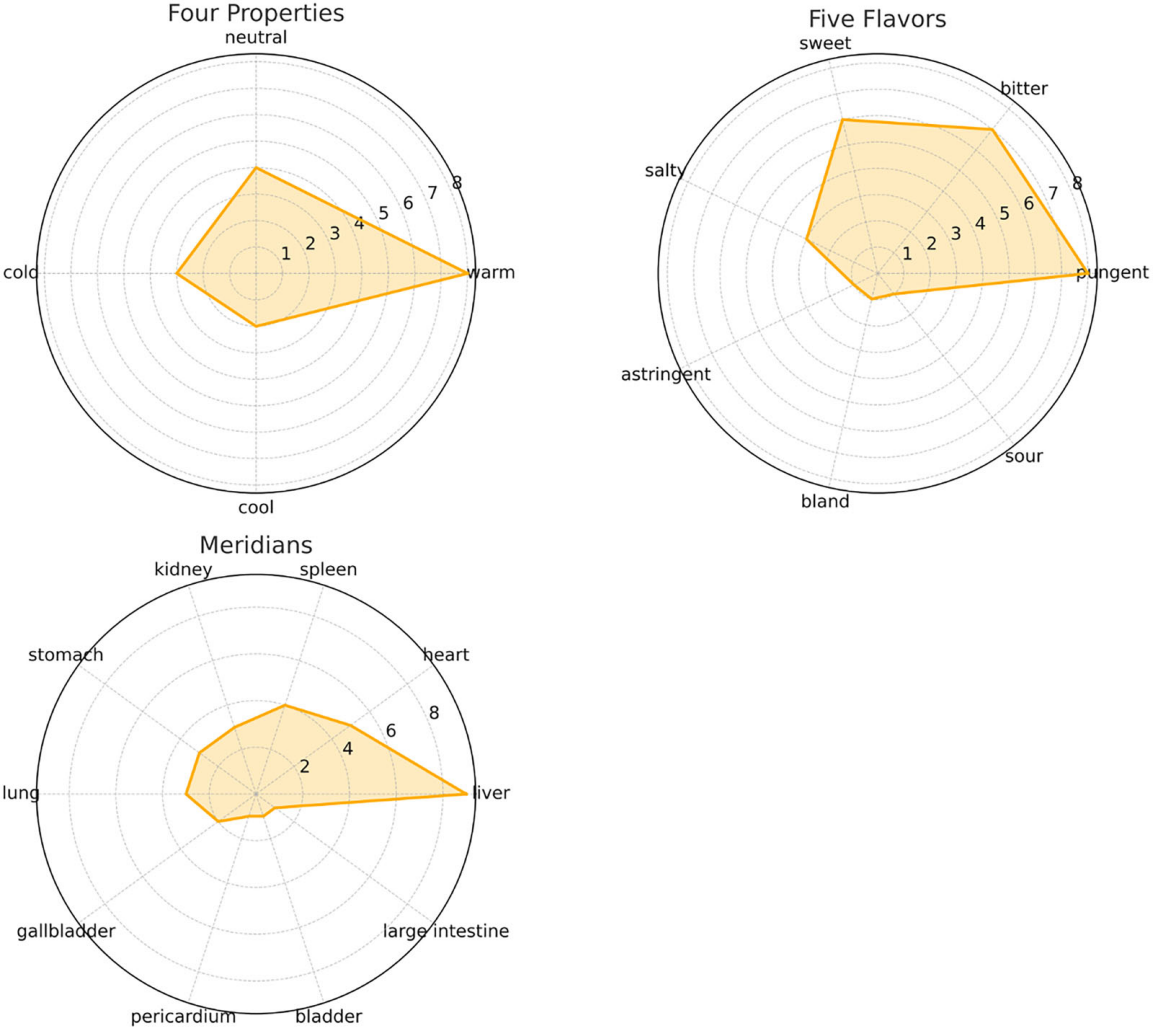
Traditional Chinese medicines for treating post‐ischemic stroke cognitive impairment: four properties, five flavors, and meridians.

According to the 2015 edition of the “Chinese Pharmacopoeia,” the properties and meridian tropism of herbs with a usage frequency greater than 10% were analyzed. These herbs included six types of properties, with warm (33.33%), neutral (25.92%), and slightly warm (18.51%) being the most common, followed by slightly cold (7.41%), cold (7.41%), and cool (7.41%). When slightly cold is combined with cold and slightly warm with warm, the frequencies of the four natures are warm (51.85%), neutral (25.93%), cold (14.81%), and cool (7.41%), with no hot properties observed. The herbs exhibited seven flavors, with pungent (29.54%), bitter (27.27%), and sweet (27.27%) being the most prevalent, followed by salty (6.82%), astringent (4.54%), bland (2.27%), and sour (2.27%). The meridian tropism involved ten organs, with the liver (30.30%), heart (16.67%), and spleen (15.15%) being the most targeted, followed by the kidney (9.09%), stomach (9.09%), lung (9.09%), gallbladder (4.54%), pericardium (3.03%), bladder (1.51%), and large intestine (1.51%), as shown in Figure 2.

### 3.4 Cluster Analysis Results

Using the classification standards of the “Thirteenth Five‐Year Plan” national textbook on “Chinese Materia Medica,” 27 herbs with a usage frequency greater than 10% (totaling 327 instances) were analyzed. These herbs were categorized based on their primary therapeutic effects. The top five categories of herbs used for treating cognitive impairment post‐ischemic stroke were blood‐activating herbs, tonifying herbs, intelligence‐enhancing herbs, wind‐calming and collaterals‐unblocking herbs, and phlegm‐resolving herbs, as shown in Table 2.

**TABLE 2:** Cluster analysis results of common Chinese medicines for treating cognitive impairment post‐ischemic stroke.

Category	Number	Type	Medicines	Effects
Category 1	81	Activating Medicines	*Ligusticum chuanxiong, Carthamus tinctorius, Prunus persica, Panax notoginseng*	Blood‐activating and stasis‐removing
			*Salvia miltiorrhiza, Curcuma aromatica, Paeonia lactiflora*	Cool blood and activate blood
			*Hirudo medicinalis*	Activate blood circulation
Category 2	81	Tonifying Medicines	*Glycyrrhiza uralensis, Astragalus membranaceus, Panax ginseng*	Tonify Qi as the main effect
			*Angelica sinensis, Rehmannia glutinosa, Polygonum multiflorum*	Tonify blood as the main effect
Category 3	42	Mental‐Enhancing Medicines	*Acorus tatarinowii*	Mainly opens orients and awakens
			*Polygala tenuifolia*	Mainly removes phlegm and opens orients
Category 4	35	Wind‐Extincting and Collateral‐Activating Medicines	*Buthus martensi, Polygonatum sibiricum, Gastrodia elata*	Mainly extinguish wind and stop spasm
			*Bombyx mori*	Mainly dispels phlegm and dissipates nodes
Category 5	21	Phlegm‐Transforming Medicines	*Paris polyphylla*	Mainly dispels wind and transforms phlegm
			*Pinellia ternata, Citrus reticulata*	Mainly invigorates the spleen and transforms phlegm

### 3.5 Association Rule Analysis

**FIGURE 3:**
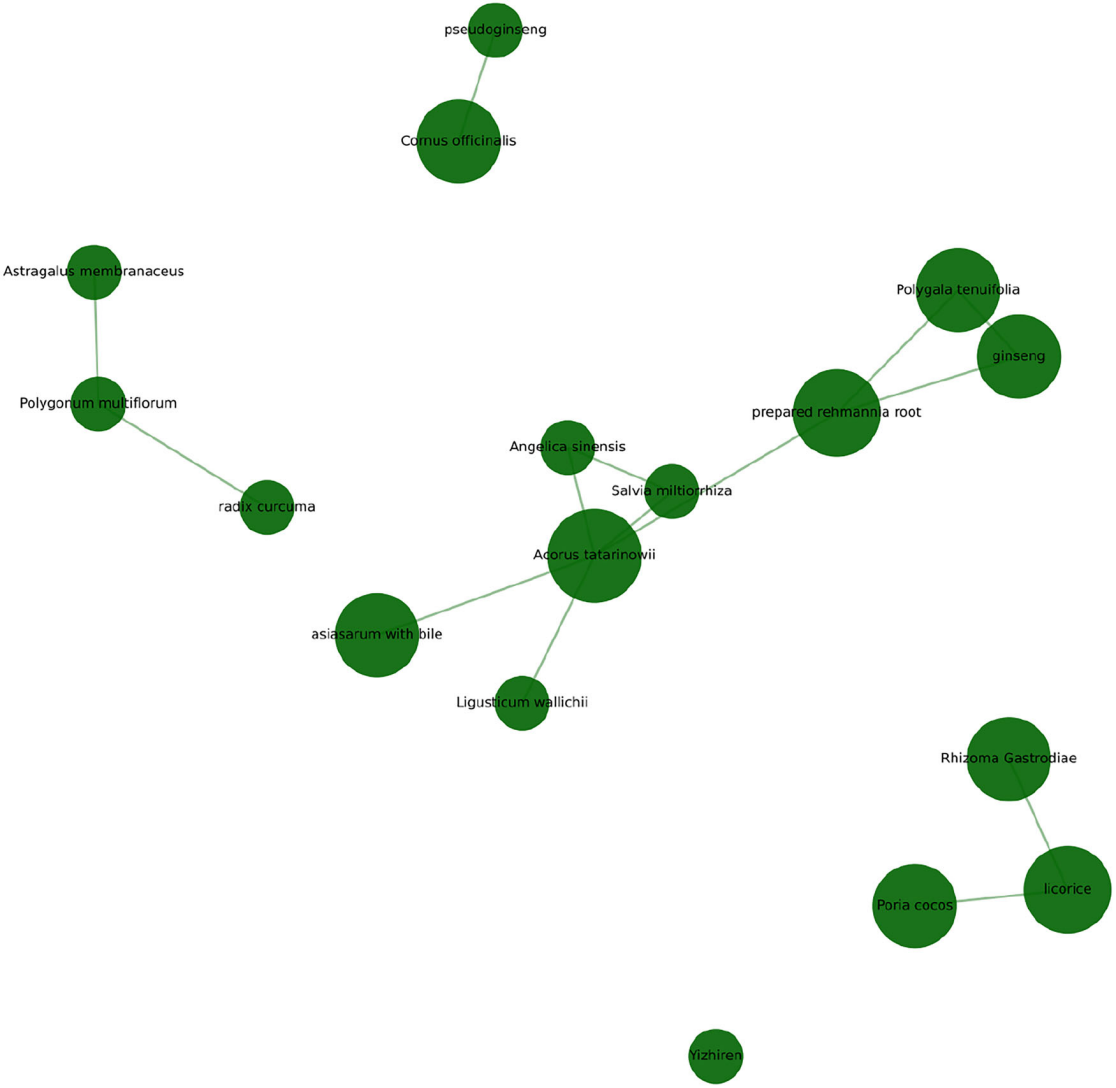
Network analysis of interactions among medications.

Using Python, an association rule analysis was conducted on the herbs within the formulas. Due to the strong correlations among the herbs, the minimum support was set at 10%, the minimum confidence at 50%, and the maximum antecedent size at 5. The top 30 association rules are presented in Table 3 and Figure 3.

**TABLE 3:** Association rules for medicinal herbs in the treatment of cognitive impairment.

Rule	Support	Confidence	Lift
*Acorus tatarinowii → Ligusticum chuanxiong*	0.26786	0.56	1.11
*Polygala tenuifolia → Acorus tatarinowii*	0.25	0.93	1.94
*Salvia miltiorrhiza → Ligusticum chuanxiong*	0.25	0.58	1.17
*Acorus tatarinowii → Polygala tenuifolia*	0.25	0.52	1.94
*Ligusticum chuanxiong → Salvia miltiorrhiza*	0.25	0.5	1.17
*Carthamus tinctorius → Salvia miltiorrhiza*	0.19643	0.69	1.6
*Angelica sinensis → Ligusticum chuanxiong*	0.19643	0.69	1.38
*Polygonatum sibiricum → Salvia miltiorrhiza*	0.18	0.91	2.12
*Polygonatum sibiricum + Ligusticum chuanxiong → Salvia miltiorrhiza*	0.16	1	2.33
*Polygonatum sibiricum + Salvia miltiorrhiza → Ligusticum chuanxiong*	0.16	0.9	1.8
*Polygonatum sibiricum → Ligusticum chuanxiong*	0.16	0.82	1.64
*Polygonatum sibiricum → Salvia miltiorrhiza + Ligusticum chuanxiong*	0.16	0.82	3.27
*Rehmannia glutinosa → Polygala tenuifolia*	0.16071	0.75	1.56
*Paeonia lactiflora → Ligusticum chuanxiong*	0.16071	0.64	1.29
*Salvia miltiorrhiza + Ligusticum chuanxiong → Polygonatum sibiricum*	0.16071	0.64	3.27
*Astragalus membranaceus → Ligusticum chuanxiong*	0.14	0.8	1.6
*Carthamus tinctorius + Ligusticum chuanxiong → Salvia miltiorrhiza*	0.14286	0.73	1.7
*Carthamus tinctorius + Salvia miltiorrhiza → Ligusticum chuanxiong*	0.14286	0.73	1.45
*Glycyrrhiza uralensis → Salvia miltiorrhiza*	0.14286	0.57	1.33
*Salvia miltiorrhiza + Ligusticum chuanxiong → Carthamus tinctorius*	0.14286	0.57	2
*Polygala tenuifolia → Glycyrrhiza uralensis*	0.14286	0.53	1.07
*Angelica sinensis → Polygala tenuifolia*	0.14286	0.5	1.04
*Carthamus tinctorius → Angelica sinensis*	0.14286	0.5	1.75
*Angelica sinensis → Carthamus tinctorius*	0.14286	0.5	1.75
*Carthamus tinctorius → Salvia miltiorrhiza + Ligusticum chuanxiong*	0.14286	0.5	2
*Salvia miltiorrhiza → Glycyrrhiza uralensis*	0.14286	0.33	1.33
*Salvia miltiorrhiza → Carthamus tinctorius + Ligusticum chuanxiong*	0.14286	0.33	1.7
*Polygala tenuifolia → Carthamus tinctorius*	0.14286	0.3	1.04
*Ligusticum chuanxiong → Polygala tenuifolia*	0.14286	0.29	1.07
*Ligusticum chuanxiong → Astragalus membranaceus*	0.14286	0.29	1.6

## 4 Discussion

This study systematically reviewed and analyzed the utilization of TCM in treating cognitive impairment following ischemic stroke. The analysis of 56 identified TCM formulas revealed a diverse array of 114 medicinal herbs, with 27 herbs showing usage frequencies exceeding 10%. Key herbs such as *Ligusticum chuanxiong*, *Acorus tatarinowii*, *Salvia miltiorrhiza, Carthamus tinctorius*, and *Angelica sinensis* were prominently featured. Association rule mining further highlighted significant herb combinations, underscoring prevalent synergies in TCM formulations aimed at cognitive recovery post‐stroke.

### 4.1 Traditional Chinese Medicine Perspectives on Post‐Ischemic Stroke Cognitive Impairment

In TCM diagnostics, post‐ischemic stroke cognitive impairment is not specifically recognized and is generally categorized under “forgetfulness” or “amnesia.” Qing Dynasty physician Wang Ang mentioned in “Materia Medica for Prepared Medicines” that memory resides in the brain and that memory loss in the elderly is due to brain depletion. In “Medical Heart Manual: Amnesia Chapter,” it is stated that “the kidney governs intelligence; when the kidney is deficient, intelligence is insufficient, hence forgetfulness of previous words.” “Treatise on Febrile Diseases” notes that those who are prone to forgetfulness must have blood stasis. “Complete Works of Jingyue” says that blood stasis in the heart leads to amnesia. The “Secret Record of the Stone Chamber” describes a “miraculous method for treating stupidity, which involves treating phlegm to cure stupidity.” In “Records of Differentiation and Treatment,” Chen Shize introduced the concept of “disease of stupidity,” attributing its primary mechanism to liver depression attacking the spleen, stomach decline causing phlegm production, phlegm accumulating in the chest, obstructing the heart orifices, damaging the spirit, depleting marrow, and brain decay. He proposed resolving depression and phlegm, and strengthening the spleen and promoting qi as the main treatment methods. Tang Rongchuan's “Theory of Blood Syndromes” states that “when blood is in the upper part, it becomes turbid and obscures clarity,” and that “blood stasis in the heart causes amnesia.” He also mentioned that “if phlegm is not resolved, the orifices will not open; if stasis is not removed, the mind cannot be controlled.” Therefore, phlegm and blood stasis are both pathological products and pathogenic factors that often coexist, and their obstruction of the orifices is the primary contradiction in this condition.

### 4.2 Analysis of Medication Frequency

From the medication frequency analysis, it is evident that there are commonalities in the use of TCM for treating post‐ischemic stroke cognitive impairment. The most frequently used herb is Chuanxiong (*Ligusticum chuanxiong*), which has the functions of promoting blood circulation, dispelling wind, and relieving pain. Its warm nature and pungent fragrance allow it to move through the blood vessels, making it effective in activating blood and resolving stasis, as well as promoting qi circulation. “Materia Medica” describes it as capable of “ascending to the head and eyes,” facilitating blood circulation and clearing the brain. The second most frequent herb is Shichangpu (*Acorus tatarinowii*), known for its pungent and bitter nature that warms the blood vessels, dispels phlegm, and awakens the mind, making it effective in treating phlegm‐dampness obstructing the orifices. Recent clinical studies have confirmed its neuroprotective effects and its significant role in treating ischemic brain injury (Qin et al. 2021). The third most frequent herb is Danshen (*Salvia miltiorrhiza*), which promotes blood circulation, removes blood stasis, and alleviates pain. It has the dual functions of activating blood and nourishing blood. “Gynecology Theory” mentions Danshen's ability to “nourish and generate blood,” comparing its efficacy to that of Peony for blood regulation and stasis removal, enhancing its therapeutic effect on ischemic stroke by improving hemorheology and platelet function (Wu et al. 2022). Studies have shown that Danshen exerts multiple protective effects in ischemic stroke by inhibiting apoptosis, reducing inflammation, and protecting neurons, thereby synergistically treating ischemic stroke (X Liu et al. 2022).

### 4.3 Analysis of Medicinal Properties and Meridian Tropism

The analysis of the medicinal properties and meridian tropism of frequently used herbs reveals that the primary properties are warm, neutral, and slightly warm, indicating that the treatment of post‐ischemic stroke cognitive impairment mainly involves warming the meridians and tonifying qi and blood. This aligns with the principle that the disease is characterized by deficiency and excess, justifying the use of blood‐activating and tonifying herbs. The flavors predominantly include pungent, bitter, and sweet. Pungent flavors disperse and move, promoting blood circulation and resolving stasis; bitter flavors clear heat, dry dampness, and drain fire while preserving yin; sweet flavors tonify, harmonize, and moderate. The herbs primarily target the liver, heart, and spleen meridians. The liver stores blood and regulates blood volume, the heart governs the blood vessels and circulates blood, ensuring the liver has sufficient blood storage. The spleen governs transformation and transportation; if the spleen's function is impaired, phlegm is generated internally, leading to phlegm obstructing the orifices and causing cognitive impairment.

### 4.4 Cluster Analysis Results

Cluster analysis indicates that the common herbs used in clinical practice for treating post‐ischemic stroke cognitive impairment are primarily blood‐activating, tonifying, intelligence‐enhancing, wind‐calming and collaterals‐unblocking, and phlegm‐resolving herbs. This classification largely corresponds with their clinical efficacy, although some categorizations can be optimized: Sanqi (*Panax notoginseng*) and Chishao (*Paeonia lactiflora*) should be classified as blood‐activating herbs. Shichangpu (*Acorus tatarinowii*) and Yuanzhi (*Polygala tenuifolia*) should be classified as intelligence‐enhancing herbs. Chenpi (*Citrus reticulata*) should be classified as a phlegm‐resolving herb.

This classification is reasonable based on literature: Sanqi is described in “Materia Medica” as bitter and warm, capable of transforming blood stasis. “Shennong's Herbal Classic” notes its effectiveness in eliminating blood stasis. Chishao is noted for its blood‐activating properties. Shichangpu is described in “Shennong's Herbal Classic” as descending qi, opening the mind, and benefiting intelligence, making it a key herb for unblocking the heart and spleen meridians. Yuanzhi is described in “Shennong's Herbal Classic” as benefiting wisdom and intelligence, calming the mind, and preventing forgetfulness. Chenpi, with its pungent, warm, and aromatic properties, enters the spleen and stomach meridians, drying dampness and awakening the spleen, often used with Banxia to resolve phlegm and regulate qi, following the principle that “to treat phlegm, first regulate qi.”

It should be noted that the processing methods of herbs like Banxia can influence their toxicity and therapeutic effect. Our standardized approach did not differentiate between processed variants, which may limit the granularity of efficacy interpretations for such herbs.

### 4.5 Association Rule Analysis

Based on the above analysis, the common herbal pairs for treating post‐ischemic stroke cognitive impairment include Shichangpu–Chuanxiong for resolving phlegm and awakening the mind, promoting blood circulation and qi movement; Chuanxiong–Danshen for activating blood, resolving stasis, and unblocking the orifices; Shichangpu–Yuanzhi for awakening the mind, resolving phlegm, and enhancing intelligence. Other herbal pairs also primarily focus on activating blood, resolving stasis, unblocking the orifices, and enhancing intelligence, indicating that the clinical treatment strategy involves combining blood‐activating herbs with orifice‐unblocking herbs. Shichangpu and Chuanxiong, in particular, are strongly associated with other herbs, highlighting their critical role in treating post‐ischemic stroke cognitive impairment. Modern pharmacological studies support this perspective, showing that ligustrazine in Chuanxiong can dilate cerebral blood vessels, improve cerebral circulation, and protect against cerebral ischemia‐reperfusion injury (Gao et al. 2015; Peng et al. 2019). Shichangpu inhibits the activation of microglial cells and reverses neuronal apoptosis, improving cognitive dysfunction by mitigating inflammation (H. J. Liu et al. 2017). Yuanzhi extracts, such as saponins, flavonoids, and polysaccharides, have been shown to improve cognitive impairment, delay dementia, protect neurons, and have sedative effects (Zhai et al. 2018). Yuanzhi saponins can improve cognitive function by enhancing the cholinergic system in the hippocampus and cerebral cortex, reducing oxidative damage, and decreasing amyloid protein expression (Shui et al. 2017; Gu and Xu 2019). The combination of Yuanzhi and Shichangpu is commonly used in TCM for synergistic effects in brain health and cognitive enhancement, demonstrating a greater‐than‐additive effect on improving cognitive function (Li et al. 2019).

#### 4.5.1 Key Herb Pairs and Their Therapeutic Roles

To further enhance the interpretability and clinical utility of the association rule analysis, we extracted and summarized the most representative herb pairs from the top 30 association rules identified in this study. These herb pairs not only reflect frequent co‐prescriptions but also align with TCM theories and modern pharmacological evidence regarding the treatment of PSCI.

(1) *Acorus tatarinowii–Polygala tenuifolia*

This pair appeared with the highest lift (1.94) and a confidence of 0.93, indicating a strong co‐occurrence and potential synergistic effect. In TCM theory, both herbs are classified as mental‐enhancing medicines that open the orifices and resolve phlegm. *Acorus tatarinowii* warms and unblocks the meridians to awaken the mind, while *Polygala tenuifolia* disperses phlegm and calms the spirit. Pharmacological studies have shown that both herbs improve cognitive function by modulating the cholinergic system, reducing oxidative stress, and inhibiting neuronal apoptosis.

(2) *Ligusticum chuanxiong–Salvia miltiorrhiza*

This is a classic blood‐activating pair with a lift of 1.17 and confidence of 0.50. *Ligusticum chuanxiong* invigorates qi and promotes blood circulation, while *Salvia miltiorrhiza* enhances microcirculation and exerts anti‐inflammatory and antioxidant effects. Both are commonly used to address blood stasis—a key pathological factor in PSCI as recognized in TCM. Modern studies also confirm their neuroprotective roles through improved cerebral perfusion and reduced ischemia‐reperfusion injury.

(3) *Carthamus tinctorius–Salvia miltiorrhiza*

This combination is frequently used to further augment blood circulation. With a confidence of 0.69 and lift of 1.60, the pairing shows a robust clinical association. TCM views *Carthamus tinctorius* as effective in unblocking the collaterals and dispersing blood stasis, which complements the stasis‐resolving and vessel‐protective properties of *Salvia miltiorrhiza*. Together, they support improved cognitive recovery by enhancing cerebrovascular function.

(4) *Angelica sinensis–Ligusticum chuanxiong*

The lift of 1.38 and confidence of 0.69 underscore the frequent clinical use of this pair. *Angelica sinensis* nourishes blood and harmonizes circulation, while *Ligusticum chuanxiong* activates qi and resolves stagnation. This combination reflects the TCM principle of combining tonification and dispersion to simultaneously address deficiency and excess, which is critical in treating complex syndromes like PSCI.

(5) *Polygonatum sibiricum–Salvia miltiorrhiza/Ligusticum chuanxiong*

*Polygonatum sibiricum* co‐occurred frequently with both *Salvia miltiorrhiza* and *Ligusticum chuanxiong*, with a lift of 2.12 and above. This combination reflects the integration of tonifying and activating principles. *Polygonatum sibiricum* nourishes yin and boosts cognitive resilience, while the activating herbs address vascular factors, forming a dual strategy of nourishing the root and unblocking the branch.

These herb pairs, highlighted through association rule mining, exemplify core therapeutic strategies in TCM for PSCI—namely, phlegm elimination, blood activation, orifice unblocking, and mental restoration.

It is important to note that while this study identified frequently co‐occurring herb pairs, the analysis did not account for the specific compatibility ratios between herbs due to inconsistent or absent dosage reporting in the source literature. Since different ratios can influence efficacy and safety, this represents a methodological limitation and highlights the need for future research that incorporates quantitative prescription data.

## 5 Conclusion

This study comprehensively examined the utilization of TCM in the treatment of cognitive impairment following ischemic stroke through systematic literature review and computational analysis. Analysis of 56 identified TCM formulas revealed 114 distinct medicinal herbs, with 27 herbs showing usage frequencies exceeding 10%. Prominent herbs such as *Ligusticum chuanxiong*, *Acorus tatarinowii*, *Salvia miltiorrhiza*, *Carthamus tinctorius*, and *Angelica sinensis* were identified as key components in these formulations. Association rule mining highlighted significant herb combinations, illustrating prevalent synergies in TCM therapies aimed at cognitive recovery post‐stroke. These findings provide valuable insights into the complex interactions and potential therapeutic mechanisms of TCM herbs in managing PSCIs. In conclusion, this research contributes to the understanding of TCM treatment strategies for post‐ischemic stroke cognitive impairment, emphasizing the importance of tailored herbal formulations and synergistic effects in clinical practice. Further studies are warranted to validate these findings and optimize treatment protocols for enhanced patient outcomes.

## Author Contributions

**Yixia Li**: conceptualization, investigation. **Wenqi Huang**: validation, visualization. **Jing Sun**: investigation, visualization. **Wenjuan Li**: writing – review and editing, writing – original draft.

## Ethics Statement

The authors have nothing to report.

## Consent

The authors have nothing to report.

## Conflicts of Interest

The authors declare no conflicts of interest.

## Peer Review

The peer review history for this article is available at https://publons.com/publon/10.1002/brb3.70829.

## Data Availability

The data that support the findings of this study are available from the corresponding author upon reasonable request.

## References

[brb370829-bib-0001] Chinese Stroke Association Vascular Cognitive Impairment . 2024. “Chinese Guidelines for the Diagnosis and Treatment of Vascular Cognitive Impairment (2024 edition).” Zhonghua Yi Xue Za Zhi 104: 2881–2894. 10.3760/cma.j.cn112137-20240501-01024. 38866700

[brb370829-bib-0002] Gao, H. J. , P. F. Liu , P. W. Li , et al. 2015. “Ligustrazine Monomer Against Cerebral Ischemia/Reperfusion Injury.” Neural Regeneration Research 10: 832–840. 10.4103/1673-5374.156S991. 26109963 PMC4468780

[brb370829-bib-0003] Gu, Z. R. , and B. H. Xu . 2019. “Radix Polygalae Extracts Improve Memory Function and Oxidative Free Radical Metabolism in Hippocampus and Cortex of Aged Rats.” Medical Journal of the Chinese People's Armed Police Forces 11: 960–963.

[brb370829-bib-0004] Guo, C. , Y. Wang , S. Wang , S. Zhang , and X. Tai . 2023. “Effect and Mechanism of Traditional Chinese Medicine Exercise Therapy on Stroke Recovery.” Evidence‐Based Complementary and Alternative Medicine: eCAM 2023: 5507186. 10.1155/2023/5507186. 36865742 PMC9974248

[brb370829-bib-0005] Kim, J. O. , S. J. Lee , and J. S. Pyo . 2020. “Effect of Acetylcholinesterase Inhibitors on Post‐Stroke Cognitive Impairment and Vascular Dementia: A Meta‐Analysis.” PLoS ONE 15: e0227820. 10.1371/journal.pone.0227820. 32032361 PMC7006920

[brb370829-bib-0006] Klamkam, P. , R. Pagcharoenpol , T. Treesaranuwattana , et al. 2022. “A Clinical Trial of Nicergoline to Prevent Temporary Threshold Shift.” Laryngoscope Investigative Otolaryngology 7: 515–522. 10.1002/lio2.746. 35434325 PMC9008157

[brb370829-bib-0007] Kuchcinski, G. , F. Munsch , R. Lopes , et al. 2017. “Thalamic Alterations Remote to Infarct Appear as Focal Iron Accumulation and Impact Clinical Outcome.” Brain 140: 1932–1946. 10.1093/brain/awx114. 28549087 PMC6248443

[brb370829-bib-0008] Li, X. Q. , J. Q. Zhao , Y. J. Tian , et al. 2019. “Memory‐Improving Substances Basis and Mechanism of Polygalae Radix, Acori Tatarinowii Rhizoma and Its Couplet Medicines.” Chinese Journal of Experimental Traditional Medical Formulae 3: 190–199. 10.13422/j.cnki.syfjx.20190339.

[brb370829-bib-0009] Liu, H. J. , X. Lai , Y. Xu , et al. 2017. “Alpha‐Asarone Attenuates Cognitive Deficit in a Pilocarpine‐Induced Status Epilepticus Rat Model via a Decrease in the Nuclear Factor‐KappaB Activation and Reduction in Microglia Neuroinflammation.” Frontiers in Neurology 8: 661. 10.3389/fneur.2017.00661. 29312110 PMC5735142

[brb370829-bib-0010] Liu, X. , D. Xiang , W. Jin , et al. 2022. “Timosaponin B‐II Alleviates Osteoarthritis‐Related Inflammation and Extracellular Matrix Degradation Through Inhibition of Mitogen‐Activated Protein Kinases and Nuclear Factor‐KappaB Pathways In Vitro.” Bioengineered 13: 3450–3461. 10.1080/21655979.2021.2024685. 35094658 PMC8973927

[brb370829-bib-0011] Peng, T. , Y. Jiang , M. Farhan , P. Lazarovici , L. Chen , and W. Zheng . 2019. “Anti‐Inflammatory Effects of Traditional Chinese Medicines on Preclinical In Vivo Models of Brain Ischemia‐Reperfusion‐Injury: Prospects for Neuroprotective Drug Discovery and Therapy.” Frontiers in Pharmacology 10: 204. 10.3389/fphar.2019.00204. 30930774 PMC6423897

[brb370829-bib-0012] Qin, G. , Y. Dong , Z. Liu , et al. 2021. “Shen‐Zhi‐Ling Oral Liquid Ameliorates Cerebral Glucose Metabolism Disorder in Early AD via Insulin Signal Transduction Pathway In Vivo and In Vitro.” Chinese Medicine 16: 128. 10.1186/s13020-021-00540-0. 34857022 PMC8638512

[brb370829-bib-0013] Shui, Z. F. , L. J. Han , J. Gong , et al. 2017. “Protective Effect of Sengenin on Hippocampus Neuronal Mitochondria of APP/PSI Double Transgenic Rat.” Chinese Journal of Gerontology 37: 5493–5495. 10.3969/j.issn.1005-9202.2017.22.004.

[brb370829-bib-0014] Tian, J. , L. B. Hu , M. Wu , et al. 2022. “Acupuncture‐Related Therapies for the Poststroke Cognitive Impairment Patients: A Protocol for Systematic Review and Network Meta‐Analysis.” Medicine 101: e28735. 10.1097/MD.0000000000028735. 35119022 PMC8812697

[brb370829-bib-0015] Wang, Y. J. , Z. X. Li , H. Q. Gu , et al. 2020. “China Stroke Statistics 2019: A Report from the National Center for Healthcare Quality Management in Neurological Diseases, China National Clinical Research Center for Neurological Diseases, the Chinese Stroke Association, National Center for Chronic and Non‐Communicable Disease Control and Prevention, Chinese Center for Disease Control and Prevention and Institute for Global Neuroscience and Stroke Collaborations.” Stroke and Vascular Neurology 5: 211–239. 10.1136/svn-2020-000457. 32826385 PMC7548521

[brb370829-bib-0016] Wu, J. , M. Li , A. Li , and X. Ji . 2022. “Mechanism of *Salvia miltiorrhiza* Bge. For the Treatment of Ischemic Stroke Based on Bioinformatics and Network Pharmacology.” Evidence‐Based Complementary and Alternative Medicine: eCAM 2022: 1767421. 10.1155/2022/1767421. 36133785 PMC9484879

[brb370829-bib-0017] Yang, Q. , J. Liu , K. L. Huang , et al. 2022. “A Systematic Review of the Efficacy of Donepezil Hydrochloride Combined With Nimodipine on Treating Vascular Dementia.” Medicine 101: e29307. 10.1097/MD.0000000000029307. 35945739 PMC9351939

[brb370829-bib-0018] Zhai, Y. , X. Meng , Y. Luo , et al. 2018. “Notoginsenoside R1 Ameliorates Diabetic Encephalopathy by Activating the Nrf2 Pathway and Inhibiting NLRP3 Inflammasome Activation.” Oncotarget 9: 9344–9363. 10.18632/oncotarget.24295. 29507694 PMC5823646

[brb370829-bib-0019] Zhou, P. T. , L. P. Wang , M. J. Qu , et al. 2019. “Dl‐3‐N‐Butylphthalide Promotes Angiogenesis and Upregulates Sonic Hedgehog Expression After Cerebral Ischemia in Rats.” CNS Neuroscience & Therapeutics 25: 748–758. 10.1111/cns.13104. 30784219 PMC6515698

